# An Educational Evaluation of Thiel Cadavers as a Model for Teaching Suturing Skills to Dental Students during the COVID-19 Pandemic

**DOI:** 10.3390/dj10070125

**Published:** 2022-07-04

**Authors:** Michaelina Macluskey, Angela S. Anderson, Mark Gribben, Simon D. Shepherd

**Affiliations:** 1 Department of Oral Surgery, School of Dentistry, University of Dundee, Dundee DD1 4HN, UK; aanderson001@dundee.ac.uk (A.S.A.); s.d.shepherd@dundee.ac.uk (S.D.S.); 2 Oral & Maxillofacial Surgery, NHS Forth Valley, Forth Valley Royal Hospital, Stirling Road, Larbert FK5 4WR, UK; mark.gribben2@nhs.scot

**Keywords:** education, dental, cadaver, COVID-19, focus group, suture

## Abstract

Suturing is an essential skill in dentistry and not one easily acquired. The COVID-19 pandemic prompted a change to the use of Thiel cadavers and online resources with the aim of improving skill acquisition using the best model available. This study investigated the utility of the Thiel cadaver for teaching suturing skills and the potential impact of the lockdown. Fifty-seven year 4 students attended a teaching session. Student views on this teaching were explored via a questionnaire survey and qualitative data collected from a focus group. Data were analysed using an inductive approach. The response rate was 53% (30 students) for the questionnaire with 9 students participating in the focus group. Independent feedback was provided by two members of the teaching staff. Online video resources were very well received by the students with 97% agreeing that it was useful preparation. Ninety percent (90%) thought that the cadaveric model was suitable for this teaching and realistic. Positive emergent themes from the focus group centred on the use of the cadaveric model and the positive and relaxed teaching and learning environment. Staff perceived this model as superior to previously used models. There were no reported negative pandemic impacts and the cadaver model was well received.

## 1. Introduction

The ability to place sutures is an essential skill in dentistry. Both teaching and objective assessment of this skill have been a focus of interest within our school. The introduction of a checklist-based assessment of dental student suture skills improved the transparency and objectivity of such assessments [1,2]. Once learned, it is important to continually practise this skill [3]. Factors affecting suturing skill teaching and acquisition include unrealistic models, impractical models (such as pigs heads), and the inappropriateness of practising on live patients. The method of Thiel-embalming employs a technique as developed and described by W. Thiel in the early 1990s [4]. The process results in cadavers with sufficiently long durability and that retain flexible tissue, which makes them highly suited to surgical training [5,6].

The impact of the COVID-19 pandemic has significantly reduced staff–student and student–patient contact for clinical teaching. A blended learning approach with both online delivery [7] and hands-on clinical training remained a strong local focus for oral surgery teaching. With traditional practical teaching sessions in the Clinical Skills Laboratory impeded due to social distancing rules, alternative venues were explored. Redeployment and limited resources meant our traditional in-house manufactured models were not deliverable.

Crucially, any online material requires supportive practical teaching; however, this needs to be delivered in a safe environment adhering to necessary social distancing and cross infection control guidelines. The large anatomy dissection room (DR) allows the preservation of social distancing, enough cadavers to allow students to work at a reasonable distance, and full cross infection control measures without the risk of exposure to patients.

This educational evaluation explored, for the first time, a blended model of teaching suturing skills using an online movie resource and Thiel cadavers during the COVID-19 pandemic. The aim was to explore student and staff perceptions on the use of a cadaver model during the pandemic. The objectives were to determine the student’s perception of the cadaver model for teaching suturing skills, and if they felt prepared for suturing on patients. A secondary objective was to determine staff perceptions of teaching in comparison to their experience of alternative models. The last objectives were to determine if the pandemic had any impact on this teaching and what refinements could be implemented to improve the overall experience of this teaching opportunity.

## 2. Materials and Methods

This mixed methods study consisted of an online student questionnaire, a student focus group, and staff feedback. The University of Dundee Ethics Committee considered this as an educational evaluation not requiring ethical approval. In September 2021, fifty-seven 4th year dental students were provided with handouts and a short in-house video available to them on the virtual learning environment (VLE) in advance of a practical session held in the anatomy dissection theatre in October 2021. Small groups of students, in a 1:3 staff to student ratio, were shown basic suture skills, complications, and suture techniques on a range of different anatomical sites. Students were able to practice placing sutures at sequentially more challenging areas from extra-oral to anterior intra-oral proceeding to posterior intra-oral. The session lasted 90 min.

### 2.1. Part 1: Questionnaire Survey

All 4th year dentals students were invited to complete an online, voluntary, anonymous questionnaire directly following the practical teaching exercise which explored student views on both the usefulness of the online materials to prepare them for the practical teaching and their views on the Thiel cadaver as a model. The questionnaire consisted of 7 questions on the video and 6 questions on the practical session with free text comment options (Question 4, Table 1). Questions were a combination of Likert responses on a scale of 1–5 [8], dichotomous responses, and a selection of pre-determined response options. The survey link was sent to the students’ university email addresses and a reminder email was sent out 2 weeks later to encourage greater participation.

### 2.2. Part 2: Focus Group

Following questionnaire completion, and to better understand the responses and add richness to the evaluation of this teaching, students were invited by email to join a qualitative evaluation focus group. The email included a participation information leaflet and students confirmed involvement and consent by return email. The focus group was held remotely using Microsoft Teams in February 2022 and was moderated by a member of staff who was not involved in the delivery of the teaching (ASA). The discussion was led by pre-determined questions that also included questions on the impact of COVID-19 on teaching (Table 2). The proceedings were audio recorded, transcribed, and checked for accuracy. It was planned to recruit a maximum of 10 participants to the focus group to attain data saturation. A thematic analysis of the transcribed discussion was carried out by the three authors independently using an inductive approach. The results were shared with two participants from the focus group to determine that the interpretation of the data was consistent with their views.

### 2.3. Part 3: Staff Reflective Accounts

Two experienced staff (MM and SDS) delivered the practical teaching with each student having access to a single 90 min session with a staff to student ratio of 1:3. As the students had experience of the Thiel cadavers for the teaching of anatomy, local anaesthetic, and exodontia it was anticipated that the students would not have any issues with the use of this model for the teaching of suturing skills. Staff independently offered a reflective account of their perception of the utility of the cadaveric model for this teaching in comparison to previous models. Additionally, they provided their impression of the impact of the pandemic on this teaching.

### 2.4. Statistics

Students completed a 10-station observed structured clinical exam (OSCE) at the end of the academic year. One station in the OSCE was a validated checklist-based assessment of suturing skills [1]. Performance in this assessment by the 2022 cohort was compared with the 2018 and 2019 cohorts using a two-tailed *t* test with a 95% confidence interval. Data were analysed by using SPSS Statistics for Windows (IBM SPSS Statistics for Windows, Version 23.0. IBM Corp.: Armonk, NY, USA).

## 3. Results

### 3.1. Part 1: Student Questionnaire

A total of 30 of the 57 students (53%) completed the anonymous online questionnaire, of which 97% (29) agreed that the video was easy to understand and 97% thought that it was useful for and supported skills acquisition during the practical session. The majority found the video useful for learning (Table 3) and 97% would access it after the practical session. Free text comments were invited on the strengths and on the weaknesses of the video with 29 and 21 responses, respectively.

These quotes are representative of the free text responses:

Strengths

*“The slow step by step demonstration was very useful, followed by the faster full run through.”*

*“The techniques were explained well, and the movie was easy to follow. It was good to have an idea of what we were doing before we entered the DR.”*

*“The methods shown in the movie is a lot easier to understand as you can see the looping of the sutures rather than picturing it in your head with no real visual.”*

Weaknesses

*“I found the tying of the knots difficult to understand from the video but it made more sense in the practical session in the DR.”*

*“The video was very good as a starter. But perhaps more footage from the perspective of the surgeon, so you can appreciate the hand movements better.”*

For the practical session, 97% of the students understood what instruments to use and felt comfortable using them, 40% found the mechanics of tying a suture easy, whilst 43% were neutral and 13.3% found it difficult. Ninety percent (90%) found that the cadaveric model was suitable for the session and realistic. Eighty-seven percent (87%) felt confident to place a simple suture in a live setting on a patient with some support. Table 4 shows the responses to the questions about aspects of the suturing sessions that the students found difficult, mostly relating to tying of the knot and how to avoid complications. In terms of the most useful for their understanding of the hands-on practical part of placing a suture, students reported that the practical demonstration and hands-on practice on the cadaver were the most helpful (97%), followed by the video (70%), while some reported that being taught by other staff was useful (16%) and other answers (10%).

Students were asked for a short narrative account on what they felt that they had learned during the session and how they might apply it in a clinical setting. Twenty-five responses were provided, and the following quotes are representative of the feedback:

*“I feel that this session was really useful to me, and I have become more confident with suturing now. I would keep refreshing my mind with the video, so I won’t forget this technique in the longer term.”*

*“The video beforehand was ideal and then the small groups and 1:1 tutorial session with the clinicians was great. I felt comfortable asking questions and the demo given was very relaxed and overall, it was an enjoyable learning experience.”*

*“I tried several locations intraorally. This has given me a better understanding of orientation of instruments and being aware of local anatomy.”*

### 3.2. Part 2: Focus Group

Nine students, four female and five males, attended an online team meeting moderated by a member of staff (ASA) who was not involved in the delivery of this teaching. Six recurrent themes emerged from the focus group session. Those themes are represented under the following broad headings:

- Supportive materials;
- Thiel cadavers as a teaching model;
- The learning and teaching environment;
- Impact of COVID-19 on teaching;
- Potential improvements to teaching;
- Confidence.

#### 3.2.1. Supportive Materials

The responses about the supportive teaching materials were generally positive. Contributory to participant skills and knowledge acquisition was the ability to engage with the available resources at their own pace, in a repetitive step-by-step fashion. The video resource was helpful as it allowed for pause, rewind, and play as many times as necessary to prepare for the session and students recognised an improved efficiency of use in the practical sessions as a result. The available preparatory materials were of sufficient quality such that the practical processes were understandable, which reduced the time in the practical session dedicated to this learning and made it more efficient. Visualisation in advance of the session was reported as useful.

Examples:

Student 1: *“Yeah, I agree. I think the video was good because you could actually see exactly what we were going to be doing in the cadavers. So it kind of meant that the time in the cadavers wasn’t like wasted.”*

Student 3: *“I found it quite helpful and it allowed me to not only like read, so obviously we had that kind of a handout, but the video allowed me to kind of a kind of see that and then kind of put into my head what I would be doing at this session.”*

Student 3: *“… and by also having the video beforehand that allow me to watch as many times as I wanted before we went in rather than it just being shown before we were with the cadavers and only seen it once. I was able to pause it and play it kind of as I wanted for my own learning.”*

Student 4: *“Yeah, I’d imagine that the session would have taken a considerable amount of time longer than what we had …if we hadn’t had the pre-recorded material available. So, we’re able to kind of pick up the information in the skills a little bit quicker.”*

#### 3.2.2. Thiel Cadavers as a Teaching Model

The main advantages and disadvantages of this model were broadly linked with positioning challenges, anatomical variation with access challenges, and the realism of tissues. Interestingly, students identified some of these factors as both disadvantages in the nature of the model yet as advantageous for their learning. That is, the cadavers are completely supine with no capacity for positional adjustment and so students needed to move to accommodate this, which was recognised as a learning point.

Examples:

Student 3: *“Yeah, I was just trying to say that that I felt that although that was a disadvantage in sometimes you were kind of putting yourself into a contorted position to get to everywhere you needed to be that actually the benefits of what erm doing it outweighed it and that, that I actually gained so much more from that experience”.*

*“So, I feel that even though that is a disadvantage and obviously you have to and we are aware that the patient would be in that position overall is actually a very beneficial process that we did.”*

Student 6: *“Yeah. And I was actually going to say that I obviously everyone knows that I’m tiny, but and I have that problem. But there was a block to stand on and be honest with the block, and for anyone in other years, who was worried about that, like, I stood on the block and was able to have really good visual access so I could see everything really well. So I didn’t feel that that was something that hinders my visual access or anything like that. I still found it very beneficial.”*

Students were expectant of the “feel” of the tissues as they had experienced the cadavers earlier in their curriculum. The anatomy was realised as beneficial, giving realistic orientation challenges and the need to combat anatomical restrictions. The feel for the tissues at different sites was appreciated along with the need to adapt the suturing technique accordingly. Students were able to experience complications of suturing sites with fragile mucosa such as the floor of the mouth. The friable nature of this tissue allowed students to immediately realise potential real-world difficulties of tissue collapse and breakdown. Maybe as expected for relative novices, the tissue was the focus rather that poor technique.

Examples:

Student 3: *“I also wanted to say that I found that by doing it repetitively in different areas of the mouth helped me rather than just doing it once over in the same kind of area by trying in the palate and then on the floor of the mouth and the gingivae and stuff, it allows you to kind of see different areas but also kind of get really get that technique ‘because repetition is the only way that I find that I learn things is just by constantly repeating it.”*

Student 4: *“I found it quite beneficial that by working in the mouth, the confined spaces having to kind of change your angulation and think about where you’re trying to get the needle to without damaging other tissues etc. I found that was quite beneficial as well.”*

Some students felt that the session using the cadaveric model was sufficient to prepare them to transfer the skill to suturing on a patient.

Example:

Student 7: *“I feel like with the right assistance with the staff Member, I’d probably be able to feel comfortable carrying a suture out.”*

#### 3.2.3. The Teaching and Learning Environment

Participants felt that the session was well structured and held within a relaxed and supportive environment. Some students may have otherwise felt anxious or nervous and this helped them manage the session. One respondent in particular described nervousness despite having been exposed to this environment earlier in their undergraduate curriculum for anatomy, yet focussing on the task too was helpful.

Examples:

Student 7: *“Yeah, I think just this…. like from what everybody said, in my experience, I think it was a really kind of well thought out structured session from the handout to the video to the actual experience itself that one on one with the clinician asking as many questions as you want.”*

Student 6: *“I was just gonna add to I think what someone said about it being really relaxed and I think that really helped me as well. cause I had been so nervous about it that because we were in first floor clinic groups which and everyone in my group would know that I would feel nervous about that kind of thing.”*

Student 7: *“I keep coming back to the point of relax environment. But like was very relaxed and supportive environment. You could leave the session as soon like when you wanted and like until you were actually completely confident at doing the suture, you could stay as long as you wanted.”*

The relaxed environment in combination with the small group size was referred to as facilitating learning through students feeling enabled to ask questions. Students reported that the small group sizes prompted freedom to raise issues or ask questions and reduced feelings of larger group intimidation.

Examples:

Student 7: *“I like that kind of relaxed environment that you kind of had you were able to kind of ask questions that you wanted. The relaxed environment was like almost like a one on one. It was very small group so you can have had that opportunity to ask as many questions as you wanted with the clinician which I found really helpful, I would say it’s hard to describe it.”*

The small groups reduced the fallow time not being directly involved in the practical aspects and prevented students from losing interest as reportedly may happen in larger group sizes. This also allowed students to watch and learn from others in the group.

Examples:

Student 3: *“And then also do it yourself, but it wasn’t too many people that were in the group that you kind of lost interest. It was enough that you actually learned from other people.”*

Student 6: *“And I don’t know if this has been said previously or emphasized, but I thought that the length of time that we’re in with the cadavers was like a really good amount of time. I think afterwards I came out feeling confident, I didn’t feel like the session was rushed, but I also didn’t feel that was there too long that I kind of lost kind of a concentration on it. So I think that the length of time that you’d kind of had is in for was an ideal. And I think that that would be good to kind of stay that way. You had enough time to do it and get confident, but it wasn’t too long.”*

#### 3.2.4. Impact of COVID-19 on Teaching

Although there was acknowledgement that COVID-19 may have had an impact on direct clinical exposure, students reported that COVID-19 had facilitated, through the necessity of distancing and space protocols, the emergence of small group sizes which they have found globally beneficial to their teaching. In addition, the benefit of small group sizes for the suture teaching and in the clinical environment was raised again and was indeed one potential advantageous change.

Examples:

Student 3: *“And I also want to say no, in a way like I understand [Student’s] point, but I also want to say that I’m not sure if you planned on having such small groups or whether it was because of COVID-19, but I think that by having such a small group when we actually got the teaching benefit, it does more. So I’m not sure if we didn’t have COVID-19 if numbers would have been larger in those groups, but if you were to ever do this again, I would suggest to keep them the size that you had with three or four students. And I felt that if that was COVID-19 related, that actually has benefited us in terms of the actual teaching…”*

#### 3.2.5. Potential Improvements to Teaching

Responses indicated that further Thiel practice sessions, using other models and access to equipment and materials during fallow clinical time or at home, could facilitate learning. There was a desire for live demonstration so students might be able to address issues and practice.

Examples:

Student 3: *“Yeah, I remember a name. First year when we had the skulls and we got to bring them home from the anatomy lab and how beneficial that was just that when you were studying it, you were able to look at the skull and work from that and it would be kind of the same kind of principle of having that available at all times for whenever you’re studying, really.”*

Student 4: *“I think one thing we could have done as an additional extra that would never require too much moving parts would have been maybe to do a video session with the tools at home. It’s kind of just walk through….of what you need to do if you didn’t have any understanding, maybe even a drop in session. Uh, where the clinician would be on, on a video demonstrating this, the way it’s done, even quite similar to what the video has in it, but with you being able to actually ask questions and then being able to explain why they’re doing certain things if they hadn’t gone through something in particular on the video.”*

#### 3.2.6. Confidence

Confidence was a recurrent two-fold theme which included confidence in the learning and teaching environment (supported by the small group size and atmosphere) and confidence in skill acquisition, likely achieved largely through practice and repetition. Some students reported that they left the session feeling confident about suturing. Confidence was also seen to be linked with having reassuring supervision.

Examples:

Student 3: *“And I don’t know if this has been said previously or emphasized, but I thought that the length of time that we’re in with the cadavers was like a really good amount of time. I think afterwards if came out feeling confident, I didn’t feel like the session was rushed, but I also didn’t feel that was there too long that I kind of lost kind of a concentration on it.”*

Student 3: *“And I also wanted to say that there have been a few people in my group that have actually placed sutures, which has been good to even hear about their experience with a patient and although it’s been some time, I think the time is the only thing they have the distance between having that session with the cadavers and then actually being able to place a suture. You know the technique but actually the confidence kind of goes over time, but from hearing just other students doing it, and they’ve succeeded with it, it is just one of those things that builds you confidence back up”*

#### 3.2.7. General Thoughts on the Course

In general, the course was well received by the participants in the study group with no overtly negative comments.

Examples:

Student 6: *“And, and like [Student] said, the small groups where perfect and I really enjoyed that and wouldn’t change it…”*

Student 7: *“… it was very, very beneficial. So I’ve got nothing but positive things to say about this session.”*

### 3.3. Part 3: Staff Reflective Accounts

Staff reflected independently on their perception of the Thiel cadavers as a model and were satisfied that the students had prepared for the session having watched the video and were engaged and familiar with the instrumentation and tissue handling. The move to small group teaching caused by COVID-19 seemed to reinvigorate the students’ enthusiasm, and on the whole their attitude, preparation, and participation in the session were encouraging.

In comparison with the customary surgical training model developed internally using a soft silicon gingiva, the Thiel model does have some advantages. The silicon model elasticity allowed an enormous latitude of stretch. The consequence of this lack of realism is that students do not appreciate how to manipulate the gingiva and that they may readily tear it on a patient if their technique is poor. Additionally, the silicon model does have some rudimentary elements which makes the understanding of complications somewhat challenging. The bulky silicone soft tissues lingually and buccally make it an easier model for suturing than the limitations of adjacent soft tissues experienced in the Thiel cadaver.

Staff were surprised at the robustness of the cadaveric tissues as several students were able to suture the same site without significant degradation. However, some sites, such as the floor of mouth, were far more realistic in demonstrating tissue fragility. This facet allows the immediate modification of technique and potentially better understanding. The Thiel model is obviously anatomically accurate with more realistic handling properties. One highly useful aspect is the ready transition from relatively easy to more difficult suturing sites in the mouth that students would not normally experience. Students commenced the skills extra-orally on a facial skeleton to understand the technique replacing traditional flatbed suture models. An immediate move to accessible anterior oral cavity sites was possible without a change in model and, readily within the same session, to advance according to a student’s skill acquisition. The Thiel model encompasses a broad variation of opportunity for learners to progress at a pace suitable to them. In retrospect, the historical silicon version may have been inhibitory of this process.

The understanding of complication management can be explained with some clinical relevance. Complications such as gingival tearing, combating tight contact points, manoeuvring around deep bony anatomical obstruction, needle sweep angle, gathering thread, and manipulation of thread in difficult anatomical sites were all easier to explain. Tissue malleability and anatomical accuracy allow for the introduction of alternative techniques (for example, the horizontal mattress suture). Similarly, the underlying anatomy aids the demonstration of complications such as oroantral communication and its management. Whether this model transfers to live patient experience is yet to be determined; however, from initial observation it appears that this might be a smoother transition for the student.

The changes to normal practice caused by the pandemic meant smaller group sizes with more favourable staff to student ratios. Staff found this more rewarding as teaching was tailored to the individual students needs with ongoing feedback on performance and time for students to observe others before attempting the procedure themselves. However, the disadvantage was that smaller ratios meant more time spent teaching by the two staff involved. The environment of the DR was relaxed and sufficiently spacious for social distancing even when four individuals were around one cadaver. The PPE used and attention to cross infection control did not have any impact on teaching. The supine position of the cadavers mimicked the positioning that students would experience in operating theatres, so gave some insight into how they may need to use steps to allow them improved vision and access.

In summary, the staff found this a very positive experience without any real limitations on what could be achieved using the cadaver model for teaching, and without any real disadvantages caused by COVID-19.

#### Assessment

The students completed a summative OSCE in April 2022 consisting of 10 stations, 1 of which was a suturing station. The suturing assessment used in this station has been in place for several years and was last run in 2019. In comparison with the global scores of the two cohorts of students before the COVID-19 lockdown, 98% of the 2022 cohort passed the suturing station in comparison with 92% in 2019 and 97% in 2018 (Table 5). The mean and standard deviation for the scores for this station were 88.7 ± 8.9 in 2018, 89.2 ± 9.0 in 2019. and 92.6 ± 7.76 in 2022. The 2022 cohort had a higher mean score than 2018 (*p* = 0.01) and 2019 (*p* = 0.03) using a *t* test with a 95% confidence interval, suggesting that a change in the model used was not detrimental to student performance in the assessment.

## 4. Discussion

The ability to suture is an essential skill in dentistry, is a learning outcome of the General Dental Council’s curriculum in the United Kingdom [9], and is necessary for compliance with guidance for the management of patients on oral anti-coagulants [10]. Many models have been advocated for teaching suturing skills [11]. In our school, animal models were used, then flat-bed models, followed by more anatomically correct silicon models made in house, making them more affordable than the commercially available models. Other schools have developed their own models to try to simulate the restricted space of intra-oral suturing [12]. To maintain this skill, the use of videos for self-monitoring has been advocated [13]

Before the COVID-19 pandemic, dental students’ experience of surgical skills was variable across the United Kingdom [14] and self-reported confidence of surgical skills was also variable [15]. The enforced shut down across the country meant a move from a traditional patient-centred education to an online blended learning with initially no patient access, and then limited socially distanced access once restrictions were relaxed. Due to the restrictions, we were unable to provide the customary teaching of suture skills (end of the third year with a staff to student ratio of 1:8) using silicon-based models in a clinical skills laboratory. However, we were able to provide this teaching in the anatomy department later in the year using Thiel cadavers as the model and wanted to determine what students and staff felt about this COVID-19-enforced modification to teaching.

Surveys of undergraduates are an effective method of gathering information on teaching delivery and student satisfaction. There are few accounts of qualitative data for oral surgery education [16]. To gain a deeper understanding of the student perspectives on the video, the cadaveric model, and the impact of the pandemic, qualitative data were sought from students.

The use of video instruction for suturing skills is widely used in medicine [17] and for exodontia [18], and we have previously used such aids for the teaching of dental students [1]. The students were generally positive about the use of the video prior to attending the practical session, reporting that the video could be used as a revision aid prior to treating their own patients. They liked the slow step-by-step approach of the video and the fact that they could review sections of the video, such as the tying of a surgeon’s knot, which was an aspect of suturing that some reported having difficulty with when suturing on the cadaver.

The technique of embalming advocated by Thiel [4] provided an ideal simulation model for teaching surgical skills in medicine as the tissues are rendered soft and supple by this technique making them more realistic [19]. We have found these cadavers to be very realistic for the teaching of exodontia [3]; however, this was the first time that we have used them for teaching suture skills and could find no other accounts of Thiel cadavers being used for simulated suturing in dentistry. Students were generally positive about the use of the Thiel cadavers for the teaching of suture skills, with the majority reporting that they thought it was a realistic model and that they felt prepared to place a suture in a patient. Confidence with the use of the instruments was good, but there seemed to be issues relating to the tying of a surgeon’s knot, with 13% finding this aspect of the technique difficult. Students gave positive feedback on the improved staff to student ratio more in keeping with the recommendation for safe practise [20] and the supportive relaxed environment, which was also commented on by staff, changes brought about by the pandemic. The focus group gave more detailed responses echoing the positive experience of the Thiel cadavers and the positive impact on teaching brought about by the lockdown. Staff reflected that this model was superior to previously used models and more durable than expected, and smaller group sizes were well received. Performance in the OSCE was at least as good as in previous years, so there seems to be no detriment to the students caused by the impact of COVID-19.

### Limitations of this Work

The limitations of this work include the relatively small sample size and poor response to the online questionnaire. Additionally, there was no objective proof that the students accessed the video in advance. There was a small number of staff involved, which could be viewed as a positive as there was consistency of teaching across the cohort and longitudinally from year to year but could be viewed negatively as it may introduce an element of reporting bias as students may not feel able to give a frank account of their perception of this teaching. The assessment of suturing skills was not carried out on a cadaveric model but on a tabletop model to make it comparable with previous years of the assessment. We could not objectively determine whether the students could transfer this skill to the chairside, but this will be the focus of future work.

The students did report that the course was positive and prepared them well; however, we are unaware of the perceptions of previous years of the course for comparison. Whilst the Thiel cadaveric model brings numerous benefits, widespread access is not one of them. Dundee is one of the few institutions in the UK with Thiel embalmed cadavers which may allow student access. The generalisability for undergraduate dental student teaching may be low.

## 5. Conclusions

The students’ perception of the cadaver model for teaching suturing skills was positive and the majority felt that this model prepared them for suturing on patients. Staff were very positive about the use of cadavers for teaching in comparison to their experience of alternative models. Despite the negative impact of COVID-19 on clinical access and teaching, the move towards cadaveric teaching of suture skills has been generally viewed positively by the students and staff and the use of cadavers for teaching surgical skills will be developed further. Future work could overcome some of the limitations of this study by comparing the cadaveric models with the previously used silicon-based models for teaching suturing skills and objectively assessing the students on both models. The same assessment could then be used for final year students when treating their own surgical cases to determine if the cadaveric model allows the students to develop transferable skills.

## Figures and Tables

**Table 1:** The questions and coding for the student questionnaire omitting free text responses.

Questions	Score and Coding
2. I feel that the pre-course suture movie was easy to understand.	Likert score 1–5
3. I feel that the suture movie was useful for and supported my skill acquisition during the practical session.	Likert score 1–5
4. On a scale of 1–10 how useful was the movie for your learning?	1 Not useful–10 Very useful
5. Will you access the movie after your practical session?	1 = Yes, 2 = No
8. Did you look at other resource material before the practical session?	1 = Yes, 2 = No, 3 = Other
9. I understand the instruments used for suturing and feel comfortable using them.	1 = Yes, 2 = No
10. I found the mechanics of tying a suture to be...	1 = Very easy, 5 = Very difficult
11. In terms of the most useful for your understanding of the hands-on practical part of placing a suture, which were the most helpful? Please tick all that apply.	The practical demonstration on the cadaver The movie posted to the VLE Someone else has taught me I have placed sutures before Other resources
12. The Thiel cadaveric model was suitable for the session and realistic.	Likert score 1–5
13. I now feel confident that I could place a simple suture in a live setting on a patient with some support.	Likert score 1–5
14. The aspects of the Oral Surgery Suturing Course that I found difficult were (select all that apply)...	Options 1–13

Likert score 1—Strongly disagree; 2—Somewhat disagree; 3—Neither disagree nor agree; 4—Somewhat agree; 5—Strongly agree.

**Table 2:** Focus group questions.

Number	Question
1	Can you outline which aspects of the video were most useful to you and how each was useful/helped you in your learning?
2	Can you outline which aspects of the video you found least useful?
3	Can you describe how you found working with the Thiel cadavers as a model for teaching suture skills?
4	Can you outline what you think are the advantages of using the cadavers?
5	Can you outline what you think are the disadvantages of using the cadavers?
6	Did anyone have a pre-existing difficulty with the setting? If so, why?
7	In what way do you think that this teaching has prepared you to place sutures on patients?
8	How do you think the COVID-19 situation has impacted on your learning of suture skills and the teaching you may have received?
9	How do you think this teaching could be improved?
10	If another opportunity came about to use the cadavers for teaching, would you be interested and why?

**Table 3:** How useful the students (*n* = 30) found the movie for their learning on a scale of 1 to 10.

Score	No of Respondents	% Respondents
1–5	0	0
6	1	3
7	4	13
8	7	23
9	5	17
10	13	43

**Table 4:** Aspects of the Suturing Course that students (*n* = 30) found difficult.

Question	Number of Responses	% of Respondents
Nothing was difficult, everything is fine	5	17
How to hold the instruments	4	13
Understanding suture complications	5	17
Knowing how to tie the knot	8	27
Manipulating the needle holders	8	27
Manipulating the toothed tissue forceps	4	13
Do not like working on cadavers	4	13
How to position the needle	5	17
What to do with the needle when tying the knot	8	27
How to tighten the knot	5	17
How to avoid complications	11	37
Sweeping the needle through the tissues	2	6
Other	1	3

**Table 5:** Performance in the suture OSCE for the class of 2018, 2019, and 2022.

Global Score	2018 *n* = 67	2019 *n* = 60	2022 *n* = 57
Excellent	11	11	10
Good pass	20	14	20
Clear pass	25	22	17
Borderline	9	8	9
Fail	2	5	1
